# The Chronic Kidney Disease Model: A General Purpose Model of Disease Progression and Treatment

**DOI:** 10.1186/1472-6947-11-41

**Published:** 2011-06-16

**Authors:** Lori A Orlando, Eric J Belasco, Uptal D Patel, David B Matchar

**Affiliations:** 1 Assistant Professor of Medicine, Duke University, 3475 Erwin Rd, Wallace Clinic Ste #204, Durham NC, 27705, USA; 2 Duke University, DUMC 3646, Durham, NC, 27710, USA; 3 Texas Tech University, AAEC, MS 42132, Lubbock, TX, 79409, USA; 4 Duke University, DUMC 3896, Durham, NC, 27710, USA

**Keywords:** chronic kidney disease, decision model, markov model

## Abstract

**Background:**

Chronic kidney disease (CKD) is the focus of recent national policy efforts; however, decision makers must account for multiple therapeutic options, comorbidities and complications. The objective of the Chronic Kidney Disease model is to provide guidance to decision makers. We describe this model and give an example of how it can inform clinical and policy decisions.

**Methods:**

Monte Carlo simulation of CKD natural history and treatment. Health states include myocardial infarction, stroke with and without disability, congestive heart failure, CKD stages 1-5, bone disease, dialysis, transplant and death. Each cycle is 1 month. Projections account for race, age, gender, diabetes, proteinuria, hypertension, cardiac disease, and CKD stage. Treatment strategies include hypertension control, diabetes control, use of HMG-CoA reductase inhibitors, use of angiotensin converting enzyme inhibitors, nephrology specialty care, CKD screening, and a combination of these. The model architecture is flexible permitting updates as new data become available. The primary outcome is quality adjusted life years (QALYs). Secondary outcomes include health state events and CKD progression rate.

**Results:**

The model was validated for GFR change/year -3.0 ± 1.9 vs. -1.7 ± 3.4 (in the AASK trial), and annual myocardial infarction and mortality rates 3.6 ± 0.9% and 1.6 ± 0.5% vs. 4.4% and 1.6% in the Go study. To illustrate the model's utility we estimated lifetime impact of a hypothetical treatment for primary prevention of vascular disease. As vascular risk declined, QALY improved but risk of dialysis increased. At baseline, 20% and 60% reduction: QALYs = 17.6, 18.2, and 19.0 and dialysis = 7.7%, 8.1%, and 10.4%, respectively.

**Conclusions:**

The CKD Model is a valid, general purpose model intended as a resource to inform clinical and policy decisions improving CKD care. Its value as a tool is illustrated in our example which projects a relationship between decreasing cardiac disease and increasing ESRD.

## Background

Chronic kidney disease (CKD) affects 13% of the US population and its incidence is increasing with the rise in major CKD risk factors, hypertension and diabetes[1]. An expanding body of evidence indicates that early control of hypertension and diabetes and the use of angiotensin converting enzyme inhibitors (ACEIs) can reduce progression of CKD and improve outcomes of those who do progress to end stage disease (ESRD)[1-3]. As a consequence, CKD has become the focus of local and national health policy efforts to promote screening and early initiation of therapy[4-6].

Among various interventions available for CKD, multiple clinical and policy questions arise. Which interventions provide improved health outcomes, when considering potential adverse effects, uncertain effectiveness, and the likelihood that events such as myocardial infarction (MI) may be more likely than end-stage renal disease (ESRD)? Do the health benefits come at an acceptable cost? How do providers account for both the wide variety of potential interventions and the dynamic epidemiology of CKD, which involves multiple, changing risk factors and competing comorbidities?

Decision modeling can assist in health care decisions for complex diseases like CKD by simulating disease progression while accounting for uncertainties in outcomes using a range of possible scenarios. Several models have been developed to address specific issues related to CKD[7-9], but they have been designed for special purposes and are usually not publically available. As a consequence, analysts must start the modeling process from scratch with each new question. Moreover, inputs to the model such as the probability of disease progression which are conditional on patient characteristics must be re-estimated with each project. One strategy to avoid this inefficiency is to create a model sanctioned by a major governmental agency, professional organization, or other accountable entity. Another strategy is to develop a general-purpose, publicly available model. These models are flexible enough to address a wide variety of clinically-and policy-relevant questions and are freely available to researchers and clinicians. The notion is that such a model could serve to promote a more informed decision-making process by allowing for consistent evaluation of various treatment strategies, and ongoing feedback from users who are encouraged to participate in the continuous improvement of the model in a public forum.

In this paper, we describe such a general-purpose, publicly available model and illustrate its potential as a tool for CKD healthcare with an example: the impact of a treatment to reduce cardiovascular risk in individuals with CKD.

## Methods

### Overview

The model is constructed to provide, for a defined population undergoing several intervention scenarios, the likelihood of a variety of outcomes important to decision makers: death, ESRD, MIs, strokes, bone disease, and rate of CKD progression.

Though the illustration here focuses on a hypothetical treatment that reduces cardiovascular risk, the model is designed to accommodate 7 specific interventions that affect the natural history of disease. These 7 strategies include: treatment with an ACEI, treatment with an HMG-CoA Reductase Inhibitor, management by a nephrologist, control of diabetes, control of hypertension, calcium and phosphorous management, and implementation of a CKD screening program. The model allows the evaluation of interventions on either a static or dynamic population. A static population is the basis of a traditional cohort decision model that follows a predefined group of individuals over their lifetime, while a dynamic population permits individuals to enter and leave the cohort over time. Dynamic modeling is the basis of community impact models, which provide estimates for the burden of disease in people living in a given catchment area, and it provides projections for a defined time horizon. In this paper we provide the results of a hypothetical treatment for a static cohort.

To guide the development of the model, we convened a panel of health policy and nephrology experts. During group meetings we elicited two lists: (1) patient characteristics judged to influence management by virtue of being known or strongly suspected to predict natural history, effectiveness/risk of treatment, cost, or quality of life/utility; and (2) outcomes that the model should be able to produce in order to promote informed decision making. In addition, when model input values were not available in the literature, we solicited opinion from our expert panel.

### Population

Assigned population characteristics include race, age, gender, diabetes, proteinuria, hypertension, pre-existing cardiovascular disease, calcium/phosphorous abnormalities, and initial CKD stage. CKD stages are defined according to the National Kidney Foundations Practice Guidelines: stage 1 (GFR ≥90 with proteinuria or hematuria), stage 2 (GFR 60-89 with proteinuria or hematuria), stage 3 (GFR 30-59), stage 4 (GFR 15-29), stage 5 (GFR < 15)[10]. These cohort parameters may be modified to suit an individual user's need and context. For example they may be set to represent the characteristics of the CKD population of California or to 50-year-old diabetics. The default population is the United States CKD population, based upon data from the National Health and Nutrition Examination Survey (NHANES)[1] (Table 1).

**Table 1:** Cohort baseline characteristics

Variable Description	CKD Stage 1	CKD Stage 2	CKD Stage 3	CKD Stage 4	CKD Stage 5	Data Sources
Male	65.80%	56.5%	37.8%	35.9%	35.9%	[19]

White race	39.5%	48%	47.5%	43.5%	20%	[19]

Age 20-30	55.1%	16%	1.90%	1.50%	0%	[19]

Age 40-59	25.1%	34%	4.8%	1.5%	5%	[19]

Age 60-69	10.1%	23%	23.2%	30.2%	10%	[19]

Age > 69	9.70%	27%	69.70%	66.80%	85%	[19]

CKD stage	30.3%	27.2%	39.0%	2.6%	1.5%	[19]

Proteinuria	14.3%	14.3%	6.1%	42.6%	NA	[1]

Diabetes	18.9%	22.1%	16.9%	17.1%	17.1%	[20]

Hypertension	24.1%	35.3%	46.5%	51.6%	94%	[19]

Calcium/Phosphorous abnormalities	3%	15%	50%	70%	85%	[21]

Pre-existing Cardiovascular disease	10%	12.9%	17.6%	25%	60%	[17]

### Model Structure

The foundation of the model is a "natural history" Markov Monte Carlo simulation with a discrete cycle length of one month (TreeAge Pro 2007, Release 1.0.2, 2009). A Monte Carlo is a patient-level simulation, which simulates the life of one individual at a time. The advantage of this type of simulation is that it permits tracking of prior health states (past events) and uses this information to account for the development of new risk factors and events. In addition, it addresses variability in input values by estimating outputs as distributions or summary statistics such as a mean and 95% confidence interval.

**Figure 1:**
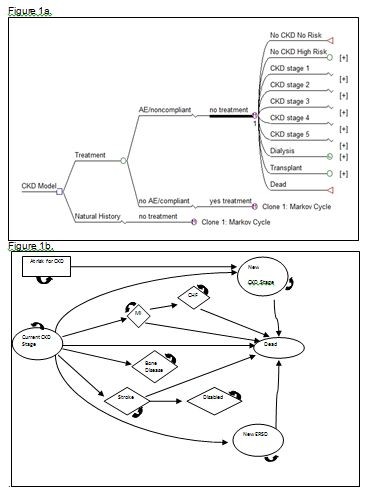
**Markov Model tree structure and monthly state diagram**. Figure 1a Markov Model tree structure up to the monthly Markov cycle. The initial decision comparison is between the natural history core and a treatment strategy. The treatment strategy arm accounts compliance with therapy by putting those who stop therapy back into the natural history core. The health states incorporated into the model are those at risk for CKD, the CKD stages, dialysis, kidney transplant, and death. Figure 1b State diagram displaying health states of the Markov monthly cycle. Each month an individual may remain in their current CKD stage, change CKD stage (either improving or declining a stage), enter dialysis or transplantation, develop bone disease, have a myocardial infarction, have a stroke from which they become disabled, have a stroke from which they are not disabled, or die. The likelihood of these events occurring are dependent upon the presence of specific covariates as well as the current CKD stage.

During each cycle, an individual may remain in their current health state or enter a new one. Health states include: CKD stages 1-5 and dialysis, bone disease, MI, congestive heart failure (CHF), stroke without disability, stroke with disability, fistula placement, transplantation, and death (Figure 1). In addition to changing health states individuals may develop comorbid conditions including diabetes, hypertension, or calcium/phosphorous abnormalities during each cycle. Once a comorbid condition develops, it remains with the patient for life and may alter future transition probabilities to other health states.

Once the GFR declines to 10 ml/min or less, either hemodialysis or kidney transplantation is initiated. Peritoneal dialysis is not included in the model since it only accounts for approximately 2% of individuals starting dialysis[11] and would further complicate the model without having any significant impact on outcomes. From here forward the term dialysis is used to mean hemodialysis only. At the time of dialysis initiation an individual may or may not have a mature fistula in place. The likelihood of having a functional fistula is set at a default value of 25% but may be modified by the user to evaluate the impact of a program implementing fistulas at higher GFRs. In the case of transplantation, the transplant may survive or fail. If it fails, the individual is assumed to revert to dialysis for the remainder of their lifespan.

### Model Parameters

#### Event Probabilities

During each monthly cycle individuals have a probability of developing calcium/phosphorous abnormalities (calcium < 8.4 mg/dL and phosphorous > 4.6 mg/dL) (Table 2) and diabetes (Table 3), as well as stage-dependent CKD complications such as hypertension (blood pressure > 135/85 mmHg) (Table 2).

**Table 2:** The monthly probability of developing CKD-related complications or conditions and the monthly change in GFR by CKD stage

Variable Description	CKD Stage 1	CKD stage 2	CKD stage 3	CKD stage 4	CKD stage 5	Data Source
Hypertension	0.08%	0.4%	0.6%	0.6%	0.6%	[22,23]

Incidence of calcium/phosphorous abnormalities	0.001%	0.005%	0.01%	0.01%	0.03%	[19]

Bone disease (in those with calcium imbalance)	0%	80%	90%	100%	100%	[19]

Stroke	0.003%	0.003%	0.003%	0.006%	0.006%	[24]

MI (no pre-existing CVD)	0.05%	0.07%	0.13%	0.13%	0.48%	[25]

MI (with pre-existing CVD)	0.27%	0.31%	0.50%	0.50%	1.00%	[25]

**Table 3:** Variables not dependent upon the current CKD stage

Variable Description	Value	Data Source
Utility of acute MI or stroke	0.1	opinion

Utility of CHF	0.76	[26]

Utility of death	0	[13]

Utility if disabled from stroke	0.35	[27]

Utility of dialysis	0.8	[13]

Utility of transplant	0.95	opinion

Monthly incidence of diabetes	0.005%	[28,29]

Probability of death from acute MI [mean (range)]	8% (1-15)	[30]

Probability of CHF after acute MI [mean (range)]	5% (1-15)	[31,32]

Probability of moderate-severe disability after stroke	33%	[33]

Probability of death from acute stroke	20%	[34]

Probability of choosing hemodialysis for renal replacement therapy	97.70%	[11]

GFR Threshold for placing fistula	30 ml/min	[11]

Monthly probability of fistula placement	25%	[11]

Monthly mortality first year on dialysis with catheter	4.25%	[35]

Monthly mortality after first year on dialysis with catheter	1.47%	[11]

Monthly mortality on dialysis with fistula	1.33%	[35]

Probability of choosing transplant for renal replacement therapy	2.30%	[11]

Mortality during transplant surgery	1.20%	[36]

Monthly probability of transplant failure	0.51%	[11]

Monthly mortality with transplant	0.31%	[11]

The probability of outcome events: bone disease, GFR change, MI, stroke, disability from stroke, CHF, cardiovascular disease-related death, and non-cardiovascular disease-related death are derived from the literature. Values for MI, stroke, bone disease, GFR change, and death are dependent upon the current CKD stage (Table 2), while the probabilities for disability from stroke, death from MI, and CHF from MI are not (Table 3). GFR change ranges from improvement to decline based upon a distribution adjusted for age, race, current CKD stage, hypertension, diabetes, proteinuria, and cardiovascular disease derived from an analysis of a primary dataset of CKD patients followed for 5 years at the Durham, NC Veteran's Affairs Hospital[12]. Being derived from a VA dataset has potential limitations in generalizeability; however it allows a detailed analysis of the normal distribution around monthly GFR changes and the specific impact of each variable, singly and combined. This level of detail is not available through other mechanisms and to explore whether the GFR change values perform appropriately we externally validate cohort GFR changes in the model against the AASK study as described in the validation section.

#### Utilities

Utilities reflect patients' preferences for health states and are used to provide an estimate of the quality of life experienced by individuals in those states. Values range from 0, defined as dead, to 1, defined as perfect health. The utilities in our simulation are derived from the literature (Table 3). Since acute MI and stroke are temporary but serious events that profoundly reduce quality of life, individuals experience lowered utilities for the month following these events. Since the post MI, post stroke, stroke-related disability, bone disease, and CHF states are permanent their utilities are applied for the remainder of the analysis.

The utility for kidney transplant was defined as the value three-fourths of the distance on the utility scale between dialysis (0.8)[13] and perfect health (1.0), or 0.95. This value was chosen since transplant has consistently been shown to have a much higher quality of life than dialysis but is not equivalent to perfect health given the immunosuppressive regimens and risk of infections[14]. In order to assess the impact of this assumption, we perform sensitivity analyses using values that range from dialysis to perfect health.

#### Treatment effects

As described above the model accommodates 7 different treatment strategies: treatment with an ACEI or ARB, treatment with an HMG-CoA Reductase Inhibitor, management by a nephrologist, control of diabetes (HgbA1c < 7.0%), control of hypertension (blood pressure < 135/85 mmHg), calcium and phosphorous management (calcium 8.4-9.5 mg/dL and phosphorous 2.7-4.6 mg/dL) and implementation of a CKD screening program. Treatment effects take into account the likelihood of being adherent with the therapy, due to either non-adherence or adverse events, and are applied as relative risk reductions on the probability of each outcome, except in the case of implementation of a screening program. In the screening strategy we simulate a dynamic cohort, which permits the addition of newly identified early stage CKD patients into the model during each cycle. This number varies based upon the characteristics of the population being screened (high risk versus low risk); however default values are derived from the KEEP study[15].

To demonstrate how the model can be used to explore the impact of treatments with a range of efficacy, we provide an example of a hypothetical treatment (TREATMENT A) that decreases the development of asymptomatic pre-clinical coronary artery and carotid artery disease by an unknown amount. In this example we show how various levels of efficacy, ranging from none to 80%, impact different endpoints, particularly development of end-stage renal disease, stroke, and MI. For simplicity, we assumed that patients were completely adherent to treatment and there were no adverse treatment events.

#### Base Case Analysis

Each Markov Monte Carlo simulation consists of 10,000 individuals, each put through the natural history and treatment comparison arms of the model. For each arm we calculate QALYs by determining the number of months spent in each health state, multiplying by the appropriate utility, summing to get quality adjusted life months and dividing by 12. Because individuals place greater value on events occurring in the present than the future, utility values are discounted by 3% per year as the cohort ages while traversing the model.

Implementing the model as a Markov Monte Carlo simulation allows tracking of specific events; in this case we track the number of MIs and strokes experienced by each individual, as well as the development of diabetes, hypertension, bone disease, ESRD, CKD progression and CHF. These values are averaged over the 10,000 individuals and compared between strategies to determine the net change (and 95% CI) attributable to the intervention.

For our example we perform a one-way sensitivity analysis of the relative risk reduction in asymptomatic pre-clinical vascular disease (RRRvasc) associated with TREATMENT A. The QALYs, MIs, strokes, and deaths are calculated using a range of values for the relative risk reduction, from 0 to 0.80. To externally validate the CKD model, we alter the model's cohort to reflect participants of two different published studies and compare the model's projections to theirs. The first study is the AASK trial[16] and the second is the study by Go et al (AS Go NEJM 2004)[17].

#### Sensitivity Analysis

In conventional sensitivity analysis, one, two, or more input values are varied over their plausible range to examine the impact on the relative preference for one intervention strategy over another. If such changes influence which strategy is preferred, or if the results of the model substantially change the relative preference of the two strategies, then the model is deemed sensitive to the variable; if the results do not change then the model is considered robust with regard to the variable and its input values. In addition to examining the relative importance of input variable on the estimated outcomes, sensitivity analysis can also be used to identify circumstances in which one strategy would be preferred over another.

In our illustration we examine each variable while also varying the relative risk reduction for asymptomatic pre-clinical vascular disease (RRRvasc), in order to assess the impact of the variable at each level of relative risk reduction.

## Results

### Validation

We compare model outputs for myocardial infarction and mortality rates to those in the Go study and GFR change to those in the AASK trial. Rates for all compared outcomes are similar. Specifically, annual myocardial infarction and mortality rates are 3.6 ± 0.9% and 1.6 ± 0.5%, respectively in the model and 4.4% and 1.6% in the Go study[17]. GFR change in the model is -3.0 ± 1.9 ml/min/year versus -1.7 ± 3.4 ml/min/year in AASK[16]. Sensitivity analyses did not alter our findings in either the baseline analysis or for the external validations.

In our base case analysis (when RRRvasc = 20% reduction), use of TREATMENT A for the primary prevention of cardiovascular disease, results in 18.2 QALYS as compared to 17.6 QALYS in the natural history arm. The lifetime risk for the US CKD cohort for MI (52% vs 75%) and cardiovascular disease mortality (5.0% vs 5.7%) is decreased by Treatment A, while the lifetime risk of stroke and stroke-related disability are unchanged and the lifetime risk of dialysis is increased (8.1% vs 7.7%). Therefore, if it is applied across the US CKD population (~ 40,000,000 individuals),[1,18] Treatment A would decrease the number of MIs by 9,200,000 and the number of cardiovascular-related deaths by 280,000 and increase the number of individuals starting dialysis by 160,000.

The lifetime risk of MI, stroke, stroke-related disability, dialysis, and cardiovascular-related death are reported across 4 levels of RRRvasc (20% to 80%) in Table 4. While MI and cardiovascular death are reduced at all levels of risk reduction, stroke and stroke-related disability are not reduced until the risk reduction reaches 60%. The mean CKD stage at death was 2.2.

**Table 4:** Model output for quality adjusted life years (QALY) and lifetime risk for dialysis, MI, stroke, disabled status, and cardiovascular disease mortality for the cohort across a range of relative risk reductions (RRR) associated with the primary prevention of cardiovascular disease (CVD)

Value of RRR for prevention of CVD	QALYs	Dialysis	MI	Stroke	Disabled	CVD Mortality
Baseline	17.6	7.7%	75%	0.7%	0.2%	5.7%

20%	18.2	8.1%	52%	0.7%	0.2%	5.0%

40%	18.5	9.0%	40%	0.7%	0.1%	3.1%

60%	19.0	10.4%	32%	0.5%	0%	2.4%

80%	19.2	11.3%	16.3%	0.2%	0%	0.8%

## Discussion

The burden of CKD is expanding rapidly as are the risk factors for developing kidney disease. Appropriate and early care can both lower the incidence of ESRD and improve the outcomes of CKD; however, the complex nature of kidney disease and the fact that it remains silent until late in its course has made implementing appropriate therapies difficult. This complexity lends itself well to decision modeling, which is designed to explore the role of competing risks and benefits within a context that incorporates patient centered values into outcomes as "quality adjusted life". For these reasons decision modeling is an ideal tool to aid decision makers and promote informed clinical and public policy. To this end we developed a general-purpose model designed to evaluate the impact of 7 different treatment strategies on outcomes including QALYs, mortality, cardiovascular disease, disease progression, and bone disease. This paper serves as an introduction to both the model and its methodology.

As a general-purpose model it is designed to be freely available for non-commercial purposes by academics and public policy analysts. Our intent is to make it as transparent as possible and to permit ongoing external validation by other users. We anticipate that this process will generate continuous feedback and that new data will continue to become available in the literature. Therefore, we designed the CKD model to be easily updated with new variable estimates and new treatment strategies on an ongoing basis using standard software, so that it may remain a useful and reliable tool.

Preliminary efforts at model validation are promising. We perform extensive sensitivity analysis as well as external validation of model outputs compared to data from cohorts not used to estimate current model inputs. We did not identify any variables that significantly changed the outcome results when varied across their plausible range. Also, external validation indicates that key model outcomes from two published studies: the AASK trial[16] and the Kaiser Cohort as reported by Go[17] are comparable to the results of the model when accounting for the characteristics of patients from those studies.

In addition to yielding comparable event rates, when comparing model results with AASK data on GFR trajectory, the results are similar. This is notable since the GFR equation is derived from a VA population and GFR modifies the risk of all outcomes. The ability to replicate the results seen in the AASK trial suggests that the model is generally applicable, providing reasonable estimates across various CKD populations, and adequately simulates the impact of blood pressure control and ACEI/ARB treatment.

As an introduction to how the model can be used and how the results may benefit decision-making, we illustrate the case of a hypothetical treatment that reduces the incidence of new cardiovascular disease. The results show how the major CKD health states interact with each other. Stroke, which can be severely disabling, is rare enough that therapies designed specifically for reduction in stroke alone will have limited effect on the population as a whole, while MI is so common that decreasing cardiac events has a tremendous impact on mortality. It also illustrates the complex nature of kidney disease and the importance of competing risks and benefits, as seen by the rise in the number of dialysis patients as cardiovascular disease declines. This has important implications for costs of care and suggests that while the prevention of cardiac disease is imperative, if implemented alone without concomitant therapies to decrease progression to ESRD is likely to increase ESRD costs.

A major limitation to decision modeling is the quality of the inputs. Inaccuracy or imprecision in the inputs propagate through the model to affect the accuracy and precision of the outputs. We address this issue of data quality, particularly those surrounding the GFR change equation by validating externally against published studies. While there are a number of covariate inputs that are not included in the model, the parameters chosen are based upon the recommendation of an expert panel and have all been shown to substantially impact at least one of the measured outcomes. As new data becomes available new parameters can be added, however increasing complexity increases the likelihood of error and does not necessarily improve accuracy. The goal of the model is to be as simple as possible while still reflecting important trends at the population level. The current covariates are all modifiable and during our sensitivity analyses performed as expected even at their extremes.

Another limitation is the level of physiologic detail. At what point are there enough health states to predict outcomes accurately, but not increase complexity unnecessarily? We limit the health states to CKD stage, ESRD on dialysis (with and without a fistula), ESRD with a kidney transplant, the presence of cardiac disease (with and without CHF), the presence of stroke (with and without moderate to severe disability), and bone disease. These states all have a significant impact on the primary outcomes of quality of life and death. As with covariates, new health states can be added as new data become available.

Our model is not designed to replace clinical trials or other primary data gathering. Its purpose is to help integrate a variety of good quality information into a form that promotes a better understanding of the implications of potential interventions, and thus more informed decision making. As demonstrated here, such a model can help identify variables that are both important and uncertain, and as such deserve the attention of clinical researchers.

## Conclusion

We developed a model designed to characterize the progression of CKD and the potential impact of a range of potentially important interventions. As a public resource we hope this will serve as a tool for decision makers and researchers, and will undergo ongoing improvement in the public domain so that the model will remain current and useful.

## Competing interests

The authors declare that they have no competing interests.

## Authors' contributions

All the authors read and approved of the final manuscript. DM assisted in the design and interpretation of the model as well as providing critical revision of this paper. LO conceived of, designed, collected, analyzed, and interpreted the data, as well as being the primary writer for this paper. EB provided critical assistance in the design and analysis of the data as well as critical revision of this paper. UP provided expertise in the conceptual design and data collection and interpretation. He also provided critical revision of this paper.

## Authors' information

DM is a leader in the decision sciences field and is the director the Duke Center for Clinical Health Policy Research and the Duke Evidence Based Practice Center for AHRQ. He was a pioneer in the field of general purpose decision models with the Stroke Policy Model upon which we based the CKD model described in this paper

LO is a senior fellow in the Duke Center for Clinical Health Policy Research and has extensive experience in decision modeling. She also assisted in the development of a decision making toolkit for providers caring for patients with CKD that was sponsored by the Renal Physician's Association.

EB is PhD in the decision sciences field.

UP is a nephrologist with extensive expertise in CKD and evaluating and collecting data for management of CKD.

## Pre-publication history

The pre-publication history for this paper can be accessed here:

http://www.biomedcentral.com/1472-6947/11/41/prepub
